# Supplementary material for: Screening and Identification of Anti-Inflammatory Compounds from Erdong Gao via Multiple-Target-Cell Extraction Coupled with HPLC-Q-TOF-MS/MS and Their Structure–Activity Relationship
Source: Molecules. 2022 Dec 30;28(1):295. doi: 10.3390/molecules28010295 (PMC9822190; doi:10.3390/molecules28010295)
Supplement: Supplementary file 1 [file molecules-28-00295-s001.zip › molecules-2075973-supplementary.pdf]

Table S1. HPLC–MS/MS data in the negative ion mode of identified in EDG

No.	t_R (min)	Identfication	$[M-H]^-$ m/z	Formula	Diff (ppm)	Fragment ions in the negative mode with the energy 50V CID
1	6.809	unknown	447.1559	$C_{30}H_{24}O_4$	9.56	447.1559, 388.1991, 285.1149, 225.0921, 165.0562, 119.0503
2	8.531	unknown	489.1621	$C_{21}H_{30}O_{13}$	-1.50	489.1621, 397.8348, 325.7311, 257.5429, 165.0581, 119.0182
3	9.037	phenethanol-*β*-vicianoside	415.1628	$C_{19}H_{28}O_{10}$	1.80	$[M-H]^-$415.1628<br>$[M-H-glc]^-$253.1944
4	20.189	unknown	285.1148	$C_{17}H_{18}O_4$	-5.48	285.1148, 267.1865, 255.0963, 183.0654, 155.8184, 119.0494
5	21.775	Ophiofurospiside I	1079.5309	$C_{47}H_{84}O_{27}$	-3.03	$[M-H]^-$1079.5309<br>$[M-H-glc]^-$917.4634<br>$[M-H-glc-rha]^-$771.4176<br>$[M-H-glc-rha-H_2O]^-$753.4068<br>$[M-H-2glc-rha-H_2O]^-$591.3511<br>$[M-H-3glc-rha-H_2O]^-$429.3023

6	22.113	Ophiopogonin L	1065.5123	$C_{50}H_{82}O_{24}$	-2.88	$[M-H]^-$ 1065.5123<br>$[M-H-xyl]^-$933.4681<br>$[M-H-glc]^-$903.4547<br>$[M-H-xyl-glc]^-$771.4294<br>$[M-H-xyl-glc-H_2O]^-$753.4184
7	22.383	Asparoside A	1079.5212	$C_{51}H_{84}O_{24}$	2.55	$[M-H]^-$1079.5212<br>[M-H-glc]-917.4739<br>[M-H-glc-rha]-771.4256;<br>[M-H-glc-rha-H_2O]-753.3938
8	23.160	(25*S*)-26-*O*-*β*-D-glucopyranosyl-5*β*-furostane-3*β*,26-diol-22α-methoxy-3-*O*-*β*-D-glucopyranosyl-(1→2)-*β*-D-glucopyranoside	979.4647	$C_{45}H_{74}O_{20}$	1.61	$[M-H+COOH]^-$979.4734<br>$[M-H]^-$933.4699<br>$[M-H-glc]^-$771.4183<br>$[M-H-glc-H_2O]^-$753.4096<br>$[M-H-2glc-H_2O]^-$591.3396<br>$[M-H-3glc-H_2O]^-$429.3081
9	23.396	Ophiofurospiside F	979.4697	$C_{45}H_{74}O_{20}$	1.36	$[M-H]^-$933.4641<br>$[M-H-rha]^-$787.4066<br>$[M-H-rha-H_2O]^-$769.3870<br>$[M-H-rha-glc-H_2O]^-$607.3527<br>$[M-H-rha-2glc-H_2O]^-$445.3106

10	26.291	Ophiopigonin K	1095.5263	$C_{51}H_{84}O_{25}$	-3.11	$[M\text{-}H]^{-}$1095.5263<br>$[M\text{-}H\text{-}H_2O]^{-}$1077.4950<br>$[M\text{-}H\text{-}H_2O\text{-}glc]^{-}$915.4415<br>$[M\text{-}H\text{-}H_2O\text{-}2glc]^{-}$753.3695<br>$[M\text{-}H\text{-}H_2O\text{-}2glc\text{-}rha]^{-}$607.5663<br>$[M\text{-}H\text{-}H_2O\text{-}3glc\text{-}rha]^{-}$445.1972
11	27.506	(25*S*)-26-*O*-*β*-D-glucopyranosyl-5*β*-furostane-5,20-diene-3*β*,26-diol-3-*O*-*α*-L-rhamnopyranosyl(1→4)-*β*-D-glucopyranoside	1061.5294	$C_{54}H_{78}O_{21}$	-4.72	$[M\text{-}H]^{-}$1061.5294<br>$[M\text{-}H\text{-}rha]^{-}$915.4752<br>$[M\text{-}H\text{-}rha\text{-}H_2O]^{-}$897.4363<br>$[M\text{-}H\text{-}2rha\text{-}H_2O]^{-}$751.4153<br>[M-H-2rha-glc-$H_2O$]-589.3372<br>$[M\text{-}H\text{-}2rha\text{-}2glc\text{-}H_2O]^{-}$427.0844
12	28.148	(25*S*)-26-*O*-*β*-D-glucopyranosyl-5*β*-furostane-22methoxy,3*β*,26-diol-3-*O*-[*α*-L-rhamnopyranosyl(1→2)-*β*-D-xylopyranosyl(1→4)]-*β*-D-glucopyranoside	1047.4923	$C_{51}H_{84}O_{22}$	-7.52	$[M\text{-}H]^{-}$1047.4923<br>$[M\text{-}H\text{-}xyl]^{-}$915.4539<br>$[M\text{-}H\text{-}xyl\text{-}rha]^{-}$769.4610<br>[M-H-xyl-rha-$H_2O$]-751.3687<br>[M-H-xyl-rha-glc-H_2O]-589.3231
13	28.621	(25S)-26-*O*-*β*-D-glucopyranosyl-5β-furostane-22methoxy,3*β*,26-diol-3-*O*-*α*-L-rhamnopyranosyl (1→6)-*β*-D-gluco-pyranoside	915.4535	$C_{46}H_{76}O_{18}$	-1.11	$[M\text{-}H]^{-}$915.4535<br>$[M\text{-}H\text{-}H_2O]^{-}$897.4897<br>$[M\text{-}H\text{-}rha\text{-}H_2O]^{-}$ 751.4037<br>$[M\text{-}H\text{-}rha\text{-}glc\text{-}H_2O]^{-}$589.3420

14	28.891	Ophiofurospiside J	917.4702	$C_{45}H_{74}O_{19}$	0.94	$[M-H]^-$917.4702<br>$[M-H-glc]^-$755.4252<br>$[M-H-2glc]^-$593.3525<br>$[M-H-3glc]^-$431.4809
15	29.228	Ophiofurospiside C	1211.5714	$C_{56}H_{92}O_{28}$	-0.96	$[M-H]^-$1211.5714<br>$[M-H-glc]^-$1049.4913<br>$[M-H-glc-xyl]^-$917.4761<br>$[M-H-glc-xyl-H_2O]^-$899.4725<br>$[M-H-glc-xyl-rha-H_2O]^-$753.1404<br>$[M-H-2glc-xyl-rha-H_2O]^-$591.4125<br>$[M-H-3glc-xyl-rha-H_2O]^-$429.5175
16	29.600	(25*R*)-26-*O*-*β*-D-glucopyranosyl(1→6)-furost-5ene-3*β*,17*α*,22*α*,26-tetrol-3-*O*-*α*-L-rhamnopyranosyl (1→2)-*O*-*β*-D-glucopyranosyl	1079.5309	$C_{51}H_{84}O_{24}$	2.41	$[M-H]^-$1079.5309<br>$[M-H-H_2O]^-$1061.5044<br>$[M-H-H_2O-glc]^-$899.4575<br>$[M-H-H_2O-glc-rha]^-$753.3976<br>$[M-H-H_2O-2glc-rha]^-$591.3551<br>$[M-H-H_2O-2glc-rha]^-$429.5311
17	30.309	ASP VII	1049.5170	$C_{50}H_{82}O_{23}$	2.75	$[M-H]^-$1049.5170;<br>$[M-H-xyl]^-$917.4718<br>$[M-H-xyl-rha]^-$771.4110<br>$[M-H-xyl-rha-H_2O]^-$753.4409<br>$[M-H-xyl-rha-glc-H_2O]^-$591.3535<br>$[M-H-xyl-rha-2glc-H_2O]^-$429.2825

18	30.781	Ophiopigonin J	1211.5714	$C_{56}H_{92}O_{28}$	-0.96	$[M-H]^-$ 1211.5714<br>$[M-H-xyl]^-$ 1079.5076<br>$[M-H-rha]^-$ 1065.4943<br>$[M-H-glc]^-$ 1049.4982<br>$[M-H-xyl-glc]^-$ 917.4617<br>$[M-H-xyl-glc-rha]^-$ 771.3982<br>$[M-H-xyl-rha-2glc]^-$ 609.3750<br>$[M-H-xyl-rha-3glc]^-$ 447.6868
19	31.288	(25S)-26-*O*-*β*-D-glucopyranosyl-5*β*-furostane-3*β*,22*α*,26-triol-12-one-3-*O*-*α*-L-rhamnopyranosyl (1→4)-*β*-D-glucopyranoside	917.4763	$C_{45}H_{74}O_{19}$	1.37	$[M-H]^-$ 917.4763<br>$[M-H-rha]^-$ 771.4251<br>$[M-H-rha-H_2O]^-$ 753.4038<br>$[M-H-rha-glc-H_2O]^-$ 591.3599<br>$[M-H-rha-glc-H_2O]^-$ 429.2953
20	31.220	(25R)-26-*O*-*β*-D-glucopyranosyl(1→4)-furost-5ene-3*β*,17*α*,22*α*,26-tetrol-3-*O*-*α*-L-rhamnopyranosyl (1→2)-*O*-*β*-D-glucopyranoside	1079.5309	$C_{51}H_{84}O_{24}$	2.5	$[M-H]^-$ 1079.5309<br>$[M-H-glc]^-$ 917.4634<br>$[M-H-glc-rha]^-$ 771.4099<br>$[M-H-glc-rha-H_2O]^-$ 753.3957<br>$[M-H-2glc-rha-H_2O]^-$ 591.3491<br>$[M-H-3glc-rha-H_2O]^-$ 429.3353
21	31.625	Ophiofurospiside F	933.4633	$C_{45}H_{74}O_{20}$	1.98	$[M-H]^-$ 933.4633<br>$[M-H-H_2O]^-$ 915.4590<br>$[M-H-2H_2O]^-$ 897.4075<br>$[M-H-rha-2H_2O]^-$ 751.3851<br>$[M-H-rha-glc-2H_2O]^-$ 589.3272<br>$[M-H-rha-2glc-2H_2O]^-$ 427.9021

22	31.693	Ophiopigonin N	1211.5685	$C_{56}H_{92}O_{28}$	1.43	$[M-H]^-$1211.5685<br>$[M-H-xyl]^-$1079.5112
23	32.064	(25*R*)-26-*O*-*β*-D-glucopyranosyl(1→6)-furost-5ene-3*β*,17*α*,22*α*,26-tetrol-3-*O*-*α*-L-rhamnopyranosyl(1→4)-*O*-*β*-D-glucopyranoside	1079.5221	$C_{51}H_{84}O_{24}$	3.68	$[M-H]^-$1079.5221<br>$[M-H-glc]^-$917.4705<br>$[M-H-glc-rha]^-$771.4135<br>$[M-H-glc-rha-H_2O]^-$753.4035<br>$[M-H-2glc-rha-H_2O]^-$591.3513<br>$[M-H-3glc-rha-H_2O]^-$429.2999
24	32.098	Ophiopogonin K’	1177.5060	$C_{56}H_{90}O_{26}$	1.05	$[M-H]^-$1177.5060<br>$[M-H+COOH]^-$1095.5263
25	33.853	Ophiofurospiside L	1049.5114	$C_{50}H_{82}O_{23}$	2.87	$[M-H]^-$1049.5114<br>$[M-H-rha-H_2O]^-$885.4546<br>$[M-H-rha-xyl-H_2O]^-$753.3936<br>$[M-H-rha-xyl-glc-H_2O]^-$591.2398<br>$[M-H-rha-xyl-2glc-H_2O]^-$429.4802
26	34.326	Ophiopogonin B	917.4581	$C_{45}H_{74}O_{19}$	4.64	$[M-H+COOH]^-$963.4841<br>$[M-H]^-$917.4581<br>$[M-H-glc]^-$755.7322<br>$[M-H-glc-rha]^-$609.5523<br>$[M-H-2glc-rha]^-$447.2838
27	35.305	Ophioponin I	1049.5075	$C_{50}H_{82}O_{23}$	-3.4	$[M-H+COOH]^-$1095.5263<br>$[M-H]^-$1049.5075<br>$[M-H-xyl]^-$917.4602<br>$[M-H-xyl-rha]^-$771.4091<br>$[M-H-xyl-rha-glc]^-$609.3462<br>$[M-H-xyl-rha-2glc]^-$447.2930

28	35.879	Ophiofurospiside M	917.4655	$C_{45}H_{74}O_{19}$	-3.77	$[M-H]^-$ 917.4655<br>$[M-H-H_2O]^-$ 899.4460<br>$[M-H-rha]^-$ 771.4094<br>$[M-H-rha-H_2O]^-$ 753.3999<br>$[M-H-rha-glc-H_2O]^-$ 591.3503<br>$[M-H-rha-2glc-H_2O]^-$ 429.2931
29	36.588	Ophiofurospiside H	1049.5175	$C_{50}H_{82}O_{23}$	-0.29	$[M-H+COOH]^-$ 1095.5232<br>$[M-H]^-$ 1049.5175<br>$[M-H-xyl]^-$ 917.4774<br>$[M-H-xyl]^-$ 917.4774<br>$[M-H-xyl-rha]^-$ 771.2968
30	36.824	Ophioponin Q	1211.5734	$C_{56}H_{92}O_{28}$	-2.61	$[M-H]^-$ 1211.5734<br>$[M-H-glc]^-$ 1049.5128
31	37.466	L-borneol-7-*O*-[*β*-D-apiofuranosyl-(1→6)]- *β*-D-glucopyranoside	447.2210	$C_{21}H_{36}O_{10}$	-5.69	$[M-H]^-$ 447.2210<br>$[M-H-glc]^-$ 285.5382
32	39.154	(22*S*)-cholest-5-ene-1*β*,3*β*,16*β*,22-tetraol-1-*O*-*α*-L-rhamnopyrnanoside-16-*O*-*β*-D-glucopyranoside	741.4370	$C_{46}H_{62}O_8$	-2.19	$[M-H]^-$ 741.4370<br>$[M-H-glc]^-$ 579.3829<br>$[M-H-glc-rha]^-$ 433.3309

33	39.289	Ophiopogoin M	1081.5468	$C_{50}H_{82}O_{25}$	-2.19	$[M-H]^-$1081.5468<br>$[M-H-glc]^-$919.5013<br>$[M-H-glc-glc]^-$757.4425
34	40.571	(25*S*)-26-*O*-*β*-D-glucopyranosyl-5*β*-furostane-3*β*,22*α*,26-triol-3-*O*-*β*-D-xylopyranosyl(1→4)-[*β*-D-glucopy-ranosyl(1→2)]-*β*-D-glucopyranoside（Officinalisnin II）	1051.5341	$C_{50}H_{84}O_{23}$	-0.99	$[M-H]^-$ 1051.5341;<br>$[M-H-xyl]^-$919.4921;<br>$[M-H-xyl-glc]^-$757.4395;<br>$[M-H-xyl-2glc]^-$595.3751;<br>$[M-H-xyl-3glc]^-$433.3540
35		25-*epi*-Officinalisnin II	1051.5384	$C_{50}H_{84}O_{23}$	-5.07	$[M-H]^-$1051.5384<br>$[M-H-xyl]^-$919.4952;<br>$[M-H-xyl-glc]^-$757.4421<br>$[M-H-xyl-2glc]^-$595.3982<br>$[M-H-xyl-3glc]^-$433.3310
36	41.483	(25S)-26-*O*-*β*-D-glucopyranosyl-5*β*-furostane-3*β*,22*α*,26-triol-3-*O*-*α*-L-rhamnopyranosyl(1→4)-[*β*-D-glucopyranosyl(1→2)]-*β*-D-glucopyranoside	1065.5491	$C_{51}H_{86}O_{23}$	1.14	$[M-H]^-$1065.5491<br>$[M-H-glc]^-$903.4978<br>$[M-H-glc-rha]^-$757.4430<br>[M-H-2glc-rha]-595.3887
37	41.685	Cixi-ophiopogon B	1047.4925	$C_{50}H_{80}O_{23}$	-2.63	$[M-H]^-$1047.4925<br>$[M-H-xyl]^-$915.4497<br>$[M-H-xyl-rha]^-$769.3965<br>$[M-H-xyl-rha-glc]^-$607.3452<br>$[M-H-xyl-rha-2glc]^-$445.4215

38	42.766	Ophiopigonin O	1063.5367	$C_{50}H_{80}O_{24}$	-3.42	$[M-H]^-$ 1063.5367<br>$[M-H-glc]^-$ 901.4731<br>$[M-H-glc-gha]^-$ 755.4165<br>$[M-H-2glc-rha]^-$ 593.3655<br>$[M-H-2glc-rha-xyl]^-$ 461.3961
39	44.082	Ophiofurospiside K	1063.5245	$C_{50}H_{80}O_{24}$	2.08	$[M-H]^-$ 1063.5245<br>$[M-H-rha]^-$ 917.4686<br>$[M-H-glc]^-$ 901.4726<br>$[M-H-glc-rha]^-$ 755.4173<br>$[M-H-2glc-rha]^-$ 593.3647<br>$[M-H-2glc-rha-xyl]^-$ 461.2117
40	44.218	(25S)-26-*O*-*β*-D-glucopyranosyl-5β-furostane-3β,22α,26-triol-3-*O*-*β*-D-glucopyranosyl(1→2)-*β*-D-glucopyranoside	919.4825	$C_{45}H_{76}O_{19}$	4.54	$[M-H]^-$ 919.4825<br>$[M-H-glc]^-$ 757.4384<br>$[M-H-2glc]^-$ 595.3910<br>$[M-H-3glc]^-$ 433.3267
41	45.500	(25*S*)-26-*O*-*β*-D-glucopyranosyl-5β-furostane-3β,22α,26-triol-3-*O*-*β*-D-glucopyranosyl (1→4)-*β*-D-glucopyranoside	919.4951	$C_{45}H_{76}O_{19}$	1.71	$[M-H]^-$ 919.4951<br>$[M-H-glc]^-$ 757.4414<br>$[M-H-2glc]^-$ 595.3980
42	54.210	(25*S*)-26-*O*-*β*-D-glucopyranosyl-5*β*-furostane-3*β*,22*α*,26-triol-3-*O*-*α*-L-rhamnopyranosyl(1→4)-[*α*-L-rhamnopyranosyl(1→2)]-*β*-D-glucopyranoside	1049.5546	$C_{51}H_{86}O_{22}$	0.47	$[M-H]^-$ 1049.5546<br>$[M-H-rha]^-$ 903.4890<br>$[M-H-2rha]^-$ 757.4714<br>$[M-H-2rha-glc]^-$ 595.3870<br>$[M-H-2rha-2glc]^-$ 433.3451

43	55.966	Ophiopogonin I'	1061.5069	$C_{51}H_{82}O_{23}$	-3.47	[M-H]$^-$1061.5069<br>[M-H-rha]$^-$915.4678<br>[M-H-glc]$^-$899.4633<br>[M-H-glc-rha]$^-$753.4092<br>[M-H-glc-rha-glc591.2696<br>[M-H-glc-rha-2glc]$^-$429.3987
44	57.417	26-*O*-*β*-D-glucopyranosyl (25*S*)-furost-5-ene-1*β*,3*β*-22*α*,26-tetraol 1-*O*-*β*-D-xylopyranosyl-(1→3)-[*α*-L-rhamnopyranosyl-(1→2)]-*β*-D-fucopyranoside	1033.4885	$C_{50}H_{82}O_{22}$	-2.83	[M-H]$^-$1033.4885<br>[M-H-xyl]$^-$901.4461<br>[M-H-rha]$^-$887.4437<br>[M-H-xyl-rha]$^-$755.3976<br>[M-H-xyl-rha-glc]$^-$593.3445<br>[M-H-xyl-rha-2glc]$^-$431.3042
45	57.485	Protodioscin	1047.5385	$C_{51}H_{84}O_{22}$	-1.5	[M-H]$^-$1047.5385<br>[M-H-rha]$^-$901.4844<br>[M-H-2rha]$^-$755.4261<br>[M-H-2rha-glc]$^-$593.3775<br>[M-H-2rha-2glc]$^-$431.3259
46	58.666	(25*S*)-26-*O*-*β*-D-glucopyranosyl-furostan-5-ene-3β,26-diol-3-*O*-*β*-D-xylopyranosyl-(1→4)-[*β*-D-glucopyranosyl-(1→2)]-*β*-D-glucopyranoside	1033.5246	$C_{49}H_{78}O_{23}$	-0.88	[M-H]$^-$1033.5246<br>[M-H-xyl]$^-$901.4807<br>[M-H-xyl-rha]$^-$755.4238<br>[M-H-xyl-rha-glc]$^-$593.3721<br>[M-H-xyl-rha-2glc]$^-$431.3137

47	58.970	(25*S*)-26-*O*-*β*-D-glucopyranosyl-5β-furostane-3*β*,22*α*,26-triol-3-*O*-α-L-rhamnopyranosyl(1→4)-[*β*-D-xylopyranosyl (1→2)]-*β*-D-glucopyranoside	1035.5381	$C_{50}H_{84}O_{22}$	-0.84	$[M-H]^-$1035.5381<br>$[M-H-xyl]^-$903.4988<br>$[M-H-xyl-rha]^-$757.4381<br>$[M-H-xyl-rha-glc]^-$595.3811<br>$[M-H-xyl-rha-glc-glc]^-$433.3308
48	59.578	(25*R*)-26-*O*-*β*-D-glucopyranosyl-furost-5-ene-3*β*,22*α*,26-triol-3-*O*-*α*-L-rhamnopyranosyl(1→2)-*O*-*β*-D-glucopyranoside	901.4724	$C_{45}H_{74}O_{18}$	-2.64	$[M-H]^-$901.4724<br>$[M-H-rha]^-$755.4198<br>$[M-H-rha-glc]^-$593.3671<br>$[M-H-rha-2glc]^-$431.3198
49	60.321	(25*S*)-26-*O*-*β*-D-glucopyranosyl-5ene-furostan-3*β*,22*α*,26-triol-3-*O*-*α*-L-rhamnopyranosyl-(1→4)-*β*-D-glucopyranoside or aspacochioside D	901.4814	$C_{45}H_{74}O_{18}$	-1.06	$[M-H]^-$901.4814<br>$[M-H-rha]^-$755.4200<br>$[M-H-rha-glc]^-$593.3880<br>$[M-H-rha-glc-glc]^-$431.0345
50	60.996	(25*S*)-26-*O*-*β*-D-glucopyranosyl-5*β*-furostan-3*β*,22*α*,26-triol-3-*O*-*α*-L-rhamnopyranosyl-(1→4)-*β*-D-glucopyranoside	903.4960	$C_{45}H_{76}O_{18}$	-1.03	$[M-H]^-$903.4960<br>[M-H-rha]-757.4386<br>[M-H-rha-glc]-595.4011<br>[M-H-rha-glc-glc]-433.3317

51	61.840	(25*S*)-26-*O*-*β*-D-glucopyranosyl-5*β*-furostan-3*β*,22*α*,26-triol-3-*O*-*α*-L-rhamnopyranosyl-(1→2)-*β*-D-glucopyranoside	903.4974	$C_{45}H_{76}O_{18}$	-1.58	$[M-H]^-$ 903.4974<br>$[M-H-rha]^-$ 757.4403<br>$[M-H-rha-glc]^-$ 595.3859<br>$[M-H-rha-glc-glc]^-$ 433.3253
52	62.751	(25*S*)-26-*O*-*β*-D-glucopyranosyl-5β-furostan-3*β*,22*α*,26-triol-3-*O*-*β*-D-xylopyranosyl-(1→4)-*β*-D-glucopyranoside	889.4820	$C_{44}H_{74}O_{18}$	-1.30	$[M-H]^-$ 889.4820<br>$[M-H-xyl]^-$ 757.4407<br>$[M-H-xyl-glc]^-$ 595.3835<br>$[M-H-xyl-glc-glc]^-$ 433.3332
53	63.123	Prazerigenin A-3-*O*-*α*-L-rhamnopyranosyl (1→2)-[*β*-D-xylopyranosyl(1→3)]-[*β*-D-glucopyranosyl(1→4)]-*β*-D-glucopyranoside	1031.4995	$C_{50}H_{78}O_{22}$	2.52	$[M-H]^-$ 1031.4995<br>$[M-H-xyl]^-$ 899.4579<br>$[M-H-xyl-rha]^-$ 753.4088<br>$[M-H-xyl-rha-glc]^-$ 591.3339
54	64.034	Pennogenin 3-*O*-*α*-L-rhamnopyranosyl (1→ 2)-[*β*-D-glucopyranosyl(1→3)]-*O*-*β*-D-glucopyranoside	899.4590	$C_{45}H_{72}O_{18}$	-3.26	$[M-H]^-$ 899.4590<br>$[M-H-rha]^-$ 753.3954<br>$[M-H-glc]^-$ 737.4073<br>$[M-H-glc-rha]^-$ 591.4958

55	65.114	Ruscogenin 3-*O*-*α*-L-rhamnopyranosyl (1→ 2)-[*β*-D-glucopyranosyl (1→3)]-*O*-*β*-D-glucopyranoside	899.4528	$C_{45}H_{72}O_{18}$	1.96	$[M-H]^-$899.4528<br>$[M-H-rha]^-$753.3734<br>$[M-H-glc]^-$737.4015<br>$[M-H-glc-rha]^-$591.3480
56	67.714	(25*R*)-26-O-β-D-glucopyranosyl-furost-5-ene-3β,22α,26-triol-3-O-[α-L-rhamnopyranosyl (1→2)-*β*-D-xylopyranosyl(1→4)]-β-D-glucopyranoside	1033.5298	$C_{50}H_{82}O_{22}$	-1.38	$[M-H]^-$1033.5298<br>$[M-H-xyl]^-$901.4834<br>$[M-H-glc-xyl]^-$739.4498
57	69.233	(25*R*)-26-O-*β*-D-glucopyranosyl-furost-5,20-diene-3β,26-diol-3-*O*-*β*-D-glucopyranosyl(1→6)-[α-L-rhamnopyranosyl(1→2)]-*O*-*β*-D-glucopyranoside	1045.5260	$C_{44}H_{86}O_{27}$	2.27	$[M-H]^-$1045.5260<br>$[M-H-glc]^-$883.4642<br>$[M-H-glc-rha]^-$737.4057<br>$[M-H-glc-rha-glc]^-$575.3561<br>$[M-H-2glc-rha-H_2O]^-$557.3468<br>$[M-H-3glc-rha-H_2O]^-$395.0246
58	69.469	(25*R*)-26-O-β-D-glucopyranosyl-furost-5-ene-3β,22α,26-triol-3-O-β-D-glucopyranosyl(1→6)-[α-L-rhamnopyranosyl(1→2)]-β-D-glucopyranoside	1053.5308	$C_{53}H_{82}O_{21}$	-3.05	$[M-H]^-$1053.5308<br>$[M-H-glc]^-$891.4694<br>$[M-H-glc-rha]^-$745.4118<br>$[M-H-2glc-rha]^-$583.3622<br>$[M-H-2glc-rha-H_2O]^-$565.3471<br>$[M-H-3glc-rha-H_2O]^-$403.4808

59	69.740	(25*S*)-26-*O*-*β*-D-glucopyranosyl-5β-furosta n-20(22)-ene-3*β*,26-diol-3-*O*-*β*-D-xylopyranosyl(1→4)-[*β*-D-glucopyranosyl(1→2)]-*β*-D-glucopyranoside	1033.4873	$C_{49}H_{78}O_{23}$	-1.15	$[M-H]^-$1033.4873<br>$[M-H-xyl]^-$901.4506<br>$[M-H-xyl-glc]^-$739.4035<br>$[M-H-xyl-2glc]^-$577.3435
60	69.807	(25S)-Spirostan-3β,17α-diol-3-O-β-D-glucopyranosyl(1→2)-[β-D-xylopyr-anosyl(1→4)]-O-β-D-glucopyranoside	887.4654	$C_{44}H_{72}O_{18}$	-0.91	$[M-H]^-$887.4654；<br>$[M-H-xyl]^-$755.3968；<br>$[M-H-xyl-glc]^-$593.3548
61	70.111	5,7,2'-trihydroxy-3'5'-dimethoxy-6,8-di methylhomoisoflavanone	373.1879	$C_{20}H_{21}O_7$	-0.9	$[M-H]^-$373.1879<br>$[M-H-B-ring-OCH_3]^-$207.0289
62	70.178	(25S)-26-O-β-D-glucopyranosyl-5β-furost an-20(22)-ene-3β,26-diol-3-O-β-D-xylopy ranosyl(1→3)-[β-D-glucopyranosyl(1→ 2)]-β-D-galactopyranoside	1033.4903	$C_{49}H_{78}O_{23}$	-0.66	$[M-H]^-$1033.4903<br>$[M-H-xyl]^-$901.4528<br>$[M-H-xyl-glc]^-$739.4050<br>$[M-H-xyl-glc-Gla]^-$577.3624
63	70.516	(25S)26-*O*-*β*-D-glucopyranosyl-5β-furost-5-ene-3β,22α,26-triol-3-*O*-*α*-L-rhamnopy-ranosyl(1→2)-[*α*-L-rhamnopyranosyl (1→4)]-β-D-glucopyranoside	1047.5383	$C_{51}H_{84}O_{22}$	-0.91	$[M-H]^-$1047.5383<br>$[M-H-rha]^-$901.4835<br>$[M-H-rha-glc]^-$739.4331<br>$[M-H-rha-2glc]^-$577.3804<br>$[M-H-2rha-2glc]^-$431.4627
64	71.259	(25*S*)26-*O*-*β*-D-glucopyranosyl-5*β*-furost-20(22)-ene-3*β*,26-diol-3-*O*-*β*-D-glucopyra nosyl(1→2)-*β*-D-glucopyranoside	901.4811	$C_{45}H_{74}O_{18}$	-1.06	$[M-H]^-$901.4811<br>$[M-H-glc]^-$739.4278<br>$[M-H-2glc]^-$577.3693

65	71.360	(25*S*)-Spirostan-3β,17α-diol-3-*O*-*β*-D-glucopyranosyl(1→2)-*O*-*β*-D-glucopyranoside	755.4265	$C_{39}H_{64}O_{14}$	-1.18	$[M-H]^-$755.4265<br>$[M-H-glc]^-$593.3680
66	72.001	(25S)26-O-β-D-glucopyranosyl-5β-furost-20(22)-ene-3β,26-diol-3-O-α-L-rhamnopyranosyl(1→4)-[β-D-xylopyranosyl (1→2)]-β-D-glucopyranoside	1017.5294	$C_{50}H_{82}O_{21}$	0.16	$[M-H]^-$1017.5294<br>$[M-H-xyl]^-$885.4877<br>$[M-H-xyl-rha]^-$793.4345<br>$[M-H-xyl-rha-glc]^-$577.3822
67	72.035	5,3',4'-trihydroxy-7-methoxy-8-methyl homoisoflavanone	329.2315	$C_{18}H_{18}O_6$	1.66	$[M-H]^-$329.2315<br>$[M-H-CO]^-$301.1138<br>$[M-H-2H_2O]^-$293.2210<br>$[M-H-B-ring]^-$211.1341
68	72.407	(25S)26-O-β-D-glucopyranosyl-5β-furost-5,20-diene-3β,26-diol-3-O-α-L-rhamnopyranosyl(1→4)-[β-D-xylopyranosyl (1→3)]-β-D-glucopyranoside	1015.5113	$C_{43}H_{84}O_{26}$	1.46	$[M-H]^-$1015.5113;<br>$[M-xyl-H]^-$883.4802;<br>$[M-xyl-rha-H]^-$737.4323;<br>$[M-xyl-rha-glc-H]^-$575.3451
69	72.474	5,7,4'-trihydroxy-3',5'-dimethoxy-8-methylhomoisoflavanone	329.233	$C_{20}H_{26}O_4$	-3.93	$[M-H]^-$329.233<br>$[M-H-H_2O]^-$311.2220<br>$[M-H-2H_2O]^-$293.2197

70	72.778	Pseudo-protodioscin	1029.5208	$C_{51}H_{82}O_{21}$	0.84	$[M-H]^-$1029.5208;<br>$[M-H-rha]^-$883.4624;<br>$[M-H-2rha]^-$737.4122;<br>$[M-H-2rha-glc]^-$575.3586
71	73.115	Ophiopogenin-3-O-α-L-rhamnpyranosyl-(1→2)-β-D-glucopyranosyl-(1→3)-β-D-glucopyranoside	915.4512	$C_{45}H_{72}O_{19}$	-2.31	$[M-H]^-$915.4512<br>$[M-H-rha]^-$769.3919<br>$[M-H-glc]^-$753.4037<br>$[M-H-glc-rha]^-$607.3326<br>$[M-H-2glc-rha]^-$445.2798
72	73.419	(25*S*)-3*β*-spirostan-3-ol-3-*O*-*β*-D-glucopyranosyl(1→2)-[*β*-D-xylopyranosyl(1→4)]-*β*-D-glucopyranoside	871.4691	$C_{44}H_{72}O_{17}$	0.66	$[M-H]^-$ 871.4691；<br>$[M-H-xyl]^-$739.4264；<br>$[M-H-xyl-glc]^-$577.3695<br>$[M-H-xyl-glc-glc]^-$415.1084
73	73.487	(25S)-26-O-β-D-glucopyranosyl-5β-furost-20(22)-en-3β,26-diol-3-O-α-L-rhamnopyranosyl(1→4)-β-D-glucopyranoside	885.4855	$C_{45}H_{74}O_{17}$	-0.2	$[M-H]^-$885.4855;<br>$[M-H-xyl]^-$739.4300;<br>$[M-H-glc]^-$577.3740
74	73.689	Ophiopogonanone G'	375.1817	$C_{19}H_{20}O_8$	1.03	$[M-H]^-$375.1817<br>$[M-H-H_2O]^-$357.1725<br>$[M-H-H_2O-CO]^-$329.5682<br>$[M-H-B-ring-C_2H_5]^-$191.0750

75	74.128	(25S)-spirostan-3β,17α-diol-12-one-3-O-β-D-glucopyranosyl(1→2)-[β-D-xylopyranosyl(1→4)]-β-D-glucopyranoside	915.4945	$C_{53}H_{72}O_{13}$	-1.97	$[M-H]^-$915.4945<br>$[M-H-glc]^-$753.4320
76	74.432	5,2'-Dihydroxy-7,8,4'-trimethoxy-6-methyl homoisoflavanone	373.1300	$C_{20}H_{22}O_7$	2.08	$[M-H]^-$373.1300<br>$[M-H-CH_3]^-$358.1077<br>$[M-H-2CH_3]^-$343.0811<br>$[M-H-B-ring-OCH_3]^-$207.0295
77	74.770	(25R)-26-O-β-D-glucopyranosyl-furost-5-ene-3β,22α,26-triol-3-O-α-L-rhamnopyra-nosyl (1→6)-O-β-D-glucopyranoside	901.4289	$C_{44}H_{70}O_{19}$	-2.18	$[M-H]^-$901.4289<br>$[M-H-xyl]^-$769.3929<br>$[M-H-xyl-rha]^-$623.3359<br>$[M-H-xyl-rha-H_2O]^-$605.3353<br>$[M-H-xyl-rha-glc-H_2O]^-$433.2753
78	75.276	Ophiopigonin R	769.4026	$C_{39}H_{62}O_{15}$	-3.23	$[M-H]^-$769.4026<br>$[M-H-xyl]^-$623.3373<br>$[M-H-xyl-glc]^-$461.2898
79	75.782	Ophiofurospiside D	949.4605	$C_{45}H_{74}O_{21}$	4.27	$[M-H]^-$949.4605<br>$[M-H-rha]^-$803.3452<br>$[M-H-rha-glc]^-$641.3524<br>$[M-H-rha-2glc]^-$479.3135
80	75.951	(25*S*)-26-*O*-*β*-D-glucopyranosyl-5*β*-furosta n-3*β*, 22*α*, 26-triol	595.3850	$C_{34}H_{44}O_9$	-3.13	$[M-H]^-$595.3850<br>$[M-H-glc]^-$433.3307

81	76.052	Cixi-ophiopogon C	753.3998	$C_{39}H_{62}O_{14}$	-2.49	$[M-H]^-$753.3998<br>$[M-H-rha]^-$607.3604<br>$[M-H-rha-glc]^-$445.2974
82	76.930	Ophiopogenin-3-O-α-L-rhamnpyranosyl-(1→2)-β-D-xylopyranosyl-(1→3)-β-D-glucopyranoside	885.4389	$C_{44}H_{70}O_{18}$	4.72	$[M-H]^-$885.4389<br>$[M-H-xyl]^-$753.3982<br>$[M-H-xyl-rha]^-$607.3376<br>$[M-H-xyl-rha-glc]^-$445.2937
83	77.403	Cixi-ophiopogon C	753.3978	$C_{39}H_{62}O_{14}$	-5.49	$[M-H]^-$753.3978<br>$[M-H-rha]^-$607.3447<br>$[M-H-rha-glc]^-$445.2936
84	77.504	(25R)-3β-[(O-β-D-glucopyranosyl (1→4)-β-D-galactopyranosyl)oxy]-5β-spirostan-12-one	753.4092	$C_{39}H_{62}O_{14}$	2.07	$[M-H]^-$753.4092<br>$[M-H-glc]^-$591.3545<br>$[M-H-glc-glc]^-$429.5092
85	77.909	Ruscogenin 1-O-α-L-rhamnopyranosyl-(1→2)-4-O-sulfo-β-D-fucopyranosido-3-O-β-D-glucopyranoside	963.4194	$C_{45}H_{72}O_{20}S$	-1.25	$[M-H]^-$963.4194<br>$[M-H-rha]^-$817.3555<br>$[M-H-glc]^-$801.3481<br>$[M-H-glc-rha]^-$655.2110<br>$[M-H-glc-rha-Fuc-SO_3H]^-$429.8510

86	79.124	(25S)-5-ene-Spirostan-3β,17α-diol-3-O-β-D-glucopyranosyl-(1→2)-O-β-D-glucopyranoside	753.4078	$C_{39}H_{62}O_{14}$	-0.58	$[M-H]^-$ 753.4078<br>$[M-H-glc]^-$ 591.3454<br>[M-H-2glc]-429.3075
87	79.597	5,7,4'-Trihydroxy-3',5'-dimethoxy-6,8-dimethylhomoisoflavanone	373.1316	$C_{20}H_{22}O_7$	-3.21	$[M-H]^-$ 373.1316<br>$[M-H-2OCH_3]^-$ 313.2354<br>$[M-H-B\text{-}ring-CH_2]^-$ 207.0667<br>$[M-H-B\text{-}ring-CH_2-CO]^-$ 207.0651
88	80.306	Ruscogenin 1-*O*-*α*-L-rhamnopyranosyl-(1→2)-4-*O*-sulfate-*α*-L-arabinopyranoside	787.3615	$C_{38}H_{60}O_{15}S$	-2.56	$[M-H]^-$ 787.3615
89	80.610	Ophiogenin 2-*O*-Acetyl-*α*-L-rhamnopyrano syl-(1→2)-O-[*β*-D-xylopyranosyl-(1→3)]-*β*-D-glucopyranoside	927.4522	$C_{46}H_{72}O_{19}$	-3.14	$[M-H]^-$ 927.4522<br>$[M-H-C_2H_2O]^-$ 885.4396<br>$[M-H-C_2H_2O-xyl]^-$ 753.4031<br>$[M-H-C_2H_2O-xyl-rha]^-$ 607.3399<br>$[M-H-C_2H_2O-xyl-rha-glc]^-$ 445.2891
90	81.015	5,4'-dihydroxy-7,3'-dimethoxy-8-methyl homoisoflavanone	343.1173	$C_{19}H_{20}O_6$	-1.49	$[M-H]^-$ 343.1173<br>$[M-H-B\text{-}ring-CH_2]^-$ 207.0687,<br>$[M-H-B\text{-}ring-C_2H_5]^-$ 193.0511,<br>$[M-H-B\text{-}ring-CH_2-CO]^-$ 179.0712

91	81.049	5,7-dihydroxy-6-methyl-3-(4′-hydroxybenzyl) chromone	297.0765	$C_{17}H_{14}O_5$	-3.89	$[M-H]^-$297.0765<br>$[M-H-B\text{-}ring-H_2O]^-$175.0408<br>$[M-B\text{-}ring-CH_2]^-$204.0450
92	81.353	Pennogenin 3-*O*-*α*-L-rhamnopyranosyl-(1→2)-*β*-D-xylopyranosyl-(1→4)-*β*-D-glucopyranoside	869.4487	$C_{37}H_{74}O_{22}$	2.73	$[M-H]^-$869.4487<br>$[M-H-xyl]^-$737.4060<br>$[M-H-rha]^-$723.3867<br>$[M-H-xyl-rha]^-$591.3589<br>$[M-H-xyl-rha-glc]^-$429.3136
93	81.758	Ophiopogonone C	353.1017	$C_{20}H_{18}O_6$	-2.19	$[M-H]^-$353.1017<br>$[M-H-CH_3]^-$338.0813<br>$[M-H-CO]^-$325.1073<br>$[M-H-2CH_3]^-$323.0823<br>$[M-H-CH_3-CO]^-$310.0809<br>$[M-H-B\text{-}ring-CH_2]^-$193.0684
94	82.062	Prazerigenin A 3-*O*-*α*-L-rhamnopyranosyl-(1→2)-*β*-D-glucopyranoside	737.4031	$C_{39}H_{62}O_{13}$	-4.00	$[M-H]^-$737.4031<br>$[M-H-rha]^-$591.3559<br>$[M-H-rha-glc]^-$429.2783
95	82.163	(25S)-5-ene-Spirostan-3β,17α-diol-3-O-α-L-rhamnopyranosyl-(1→2)-O-β-D-glucopyranoside	737.4123	$C_{39}H_{62}O_{13}$	2.5	$[M-H]^-$737.4123<br>$[M-H-rha]^-$591.3551<br>$[M-H-rha-glc]^-$429.3047

96	82.568	Ophiopogonanone E	359.1105	$C_{19}H_{20}O_7$	-3.76	$[M-H]^-$ 359.1105<br>$[M-H-CH_3]^-$ 344.0887<br>$[M-H-B\text{-}ring\text{-}CH_2]^-$ 193.0501<br>$[M-H-B\text{-}ring\text{-}CH_2\text{-}CO]^-$ 165.0702
97	82.635	Prazerigenin A-3-*O*-*α*-L-rhamnopyranosyl-(1→3)-*β*-D-xylopyranosyl-(1→4)-*β*-D-glucopyranoside	869.4487	$C_{37}H_{74}O_{22}$	2.73	$[M-H]^-$ 869.4487<br>$[M-H-xyl]^-$ 737.4060<br>$[M-H-rha]^-$ 723.3867<br>$[M-H-xyl-rha]^-$ 591.3589<br>$[M-H-xyl-rha-glc]^-$ 429.3136
98	82.771	(25*S*)-17*α*,27-dihydroxyspirost-5-en-3β-yl O-(4-*O*-acetyl-α-L-rhamnopyranosyl)-(1→2)-*β*-D-glucopyranoside	795.3923	$C_{48}H_{60}O_{10}$	0.06	$[M-H]^-$ 795.3923<br>$[M-H-Ac]^-$ 753.4002<br>$[M-H-Ac-H_2O]^-$ 735.3901
99	82.906	5,7-dihydroxy-8,4'-dimethoxy-6-methyl homoisoflavanone	343.1172	$C_{19}H_{20}O_6$	-5.49	$[M-H]^-$ 343.1172<br>$[M-H-B\text{-}ring\text{-}CH_2]^-$ 207.0658<br>$[M-H-B\text{-}ring\text{-}CH_2\text{-}CO]^-$ 179.0704
100	83.344	5,2´-dihydroxy-7,4´-dimethoxy-6,8-dimeth ylhomoisoflavanone	357.1325	$C_{20}H_{22}O_6$	-4.85	$[M-H]^-$ 357.1325<br>$[M-H-CH_3]^-$ 342.0961<br>$[M-H-2CH_3]^-$ 327.0878<br>$[M-H-B\text{-}ring\text{-}CH_2]^-$ 206.0571<br>$[M-H-B\text{-}ring\text{-}CH_2\text{-}CO]^-$ 178.0619

101	83.615	1-O-sulfate-ruscogenin	509.2530	$C_{27}H_{42}O_7S$	0.50	$[M-H]^-$ 509.2530
102	83.885	3,5-dihydroxy-7,4'-dimethoxy-6-dimethylhomoisoflavanone	343.1189	$C_{19}H_{20}O_6$	-2.49	$[M-H]^-$343.1189<br>$[M-H-H_2O-OCH_3]^-$297.1964,<br>$[M-B\text{-}ring-H_2O-CH_2]^-$207.0654,<br>$[M-B\text{-}ring-H_2O-C_3H_3O]^-$153.0553
103	84.323	5,7,2'-Trihydroxy-3',4'-methylenedioxy-6,8-dimethylhomoisoflavone	355.0815	$C_{19}H_{16}O_7$	-3.67	$[M-H]^-$355.0815<br>$[M-H-H_2O]^-$337.0683<br>$[M-H-H_2O-CO]^-$309.0711<br>$[M-H-B\text{-}ring-CH_2]^-$205.0484<br>$[M-H-B\text{-}ring-CH_2-CO]^-$177.0533
104	84.661	5-hydroxy-3',4'-dimethoxy-6,8-dimethyl homoisoflavanone	341.1018	$C_{19}H_{18}O_6$	-1.26	$[M-H]^-$341.1018<br>$[M-H-CH_3]^-$326.0785<br>$[M-H-2CH_3]^-$311.0572<br>$[M-B\text{-}ring-H_2O-CH_2]^-$207.0547<br>$[M-B\text{-}ring-2H_2O-CH_2]^-$189.0545<br>$[M-H-B\text{-}ring-CH_2-CO]^-$179.0334
105	85.066	Ophiopigonin S	723.3925	$C_{38}H_{60}O_{13}$	3.46	$[M-H]^-$723.3925<br>$[M-H-xyl]^-$591.3611<br>$[M-H-xyl-glc]^-$429.3472

106	85.302	Ophiopogonin A	911.4700	$C_{39}H_{76}O_{23}$	3.00	$[M-H]^-$ 911.4700<br>$[M-H-AC]^-$ 869.4524<br>$[M-H-AC-H_2O]^-$ 851.4407<br>$[M-H-AC-xyl]^-$ 737.4049<br>$[M-H-AC-xyl-rha]^-$ 591.3665<br>$[M-H-AC-xyl-rha-glc]^-$ 429.2992
107	85.404	(25*S*)-3*β*-[(*O*-*α*-L-rhamnopyranosyl-(1→2)-*β*-D-galactopyranosyl)oxy]-5*β*-spirostan-12-one	737.4144	$C_{39}H_{62}O_{13}$	-0.35	$[M-H]^-$ 737.4144
108	86.484	Prazerigenin A 3-*O*-[2-*O*-Acetyl-*α*-L-rhamnopyranosyl-(1→2)-*β*-D-glucopyranoside]	779.3744	$C_{41}H_{64}O_{14}$	-4.22	$[M-H]^-$ 779.3744<br>$[M-H-2H_2O]^-$ 737.4038<br>$[M-H-2H_2O-rha]^-$ 591.3275<br>$[M-H-2H_2O-rha-glc]^-$ 429.2735
109	86.653	(25S)-3β-spirostan-3-ol-3-O-β-D-glucopyranosyl-(1→2)-[β-D-xylopyranosyl-(1→4)]-β-D-glucopyranoside	871.4683	$C_{44}H_{72}O_{17}$	-2.21	$[M-H]^-$ 871.4683<br>$[M-H-xyl]^-$ 739.4220<br>$[M-H-xyl-glc]^-$ 577.3742
110	87.125	(25S)-5β-spirostan-3β-ol-3-O-α-L-rhamnopyranosyl-(1→2)-[β-D-glucopyranosyl-(1→4)]-β-D-glucopyranoside	885.4868	$C_{38}H_{78}O_{22}$	3.25	$[M-H]^-$ 885.4868<br>$[M-H-rha]^-$ 739.3975<br>$[M-H-rha-glc]^-$ 577.3876

111	87.463	Ophiopogonanone A	328.0971	$C_{18}H_{16}O_6$	-3.28	$[M-H]^-$ 237.0863<br>$[M-H-CH_3]^-$ 313.2347,<br>$[M-B-ring-CH_2]^-$ 205.0513<br>$[M-B-ring-C_2H_5]^-$ 192.0416<br>$[M-H-CH_3-H_2O]^-$ 297.0794,<br>$[M-CH_3-H_2O-B-ring-CH_2]^-$ 164.0467,<br>$[M-C_2H_5-H_2O-B-ring-CO]^-$ 136.0159
112	87.767	Prazerigenin A 3-*O*-[2-*O*-Acetyl-*α*-L-rhamnopyranosyl-(1→2)]-*β*-D-xylopyranosyl-(1→4)-*β*-D-glucopyranoside	911.4588	$C_{46}H_{72}O_{18}$	-3.43,	$[M-H]^-$ 911.4588<br>$[M-H-AC]^-$ 869.4496<br>$[M-H-AC-H_2O]^-$ 851.4404<br>$[M-H-AC-xyl]^-$ 737.4083<br>$[M-H-AC-xyl-rha]^-$ 591.3460<br>$[M-H-AC-xyl-rha-glc]^-$ 429.3096
113	88.543	5,7-dihydroxy-4'-methoxy-6-methyl homoisoflavanone	313.1062	$C_{18}H_{18}O_5$	3.02	$[M-H]^-$ 313.1062<br>$[M-H-2CH_3]^-$ 283.1251<br>$[M-H-B-ring]^-$ 207.0761<br>$[M-H-B-ring-CH_2]^-$ 192.0418<br>$[M-H-B-ring-CH_2-CO]^-$ 164.0461<br>$[M-H-B-ring-CH_2-2CO]^-$ 136.0169

114	88.847	(25S)-26-O-β-D-glucopyranosyl-5β-furostan-3β, 22α, 26-triol	595.2897	$C_{34}H_{44}O_9$	-2.61	$[M-H]^-$ 595.2897<br>$[M-H-glc]^-$ 433.2935
115	89.083	Pennogenin 3-*O*-[2-*O*-Acetyl-α-L-rhamno pyranosyl-(1→2)-β-D-glucopyranoside]	799.4160	$C_{41}H_{64}O_{14}$	-4.22	$[M-H]^-$799.4160<br>$[M-H-2H_2O]^-$737.4053<br>$[M-H-2H_2O-rha]^-$591.3284<br>$[M-H-2H_2O-rha-glc]^-$429.2935
116	89.421	(25S)-5β-spirostan-3β-ol-3-O-β-D-glucopyranosyl-(1→2)-β-D-glucopyranoside	739.4273	$C_{39}H_{64}O_{13}$	-0.28	$[M-H]^-$739.4273<br>$[M-H-glc]^-$577.3855<br>$[M-H-2glc]^-$415.3532
117	90.603	Ophiopogonin D	853.4520	$C_{44}H_{70}O_{16}$	-5.05	$[M-H]^-$853.4520<br>$[M-H-xyl]^-$721.4112<br>$[M-H-rha]^-$707.5198<br>$[M-H]-xyl-rha]^-$575.3555<br>$[M-H]-xyl-rha-Fuc]^-$413.4394
118	90.738	Methylophiopogonone A	339.0862	$C_{19}H_{16}O_6$	-2.20	$[M-H]^-$339.0862<br>$[M-H-CO]^-$311.0917<br>$[M-H-B-ring-CH_2]^-$205.0484<br>$[M-H-B-ring-CH_2-CO]^-$179.0349

119	92.156	Ophiopogonin D'	853.4505	$C_{44}H_{70}O_{16}$	1.48	$[M-H]^-$853.4505<br>$[M-H-xyl]^-$721.4119<br>$[M-H-rha]^-$707.3851<br>$[M-H]-xyl-rha]^-$575.3504<br>$[M-H]-xyl-rha-glc]^-$413.2125
120	92.358	Methylophiopogonone B	325.1110	$C_{19}H_{18}O_5$	-1.75	$[M-H]^-$325.1110<br>$[M-H-CH_3]^-$310.2103<br>$[M-H-B-ring-CH_2]^-$205.0433<br>$[M-H-B-ring-CH_2-CO]^-$179.0346
121	92.763	Methylophiopogonanone A	341.1025	$C_{19}H_{18}O_6$	1.64	$[M-H]^-$341.1025<br>$[M-H-2CH_3-CO]^-$ 283.1336,<br>$[M-B-ring]^-$220.0710,<br>$[M-H-B-ring-CH_2]^-$206.0567,<br>$[M-H-B-ring-CH_2-CO]^-$ 178.0626
122	94.755	Methylophiopogonanone B	327.1228	$C_{19}H_{20}O_5$	-3.04	$[M-H]^-$327.1228<br>$[M-H-B-ring-CH_2]^-$206.0581,<br>$[M-H-B-ring-CH_2-CO]^-$178.0621
123	95.869	Ophiopogonin B	721.409	$C_{39}H_{62}O_{12}$	-3.56	$[M-H+COOH]^-$767.4249<br>$[M-H]^-$721.4096<br>$[M-H-rha]^-$575.3523
124	96.274	Ophiopogonanone D	369.0944	$C_{20}H_{18}O_7$	1.66	$[M-H]^-$369.0944<br>$[M-H-CH_3]^-$354.0760<br>$[M-H-B-ring-CH_2]^-$205.0645<br>$[M-H-B-ring-CH_2-CO]^-$179.0316

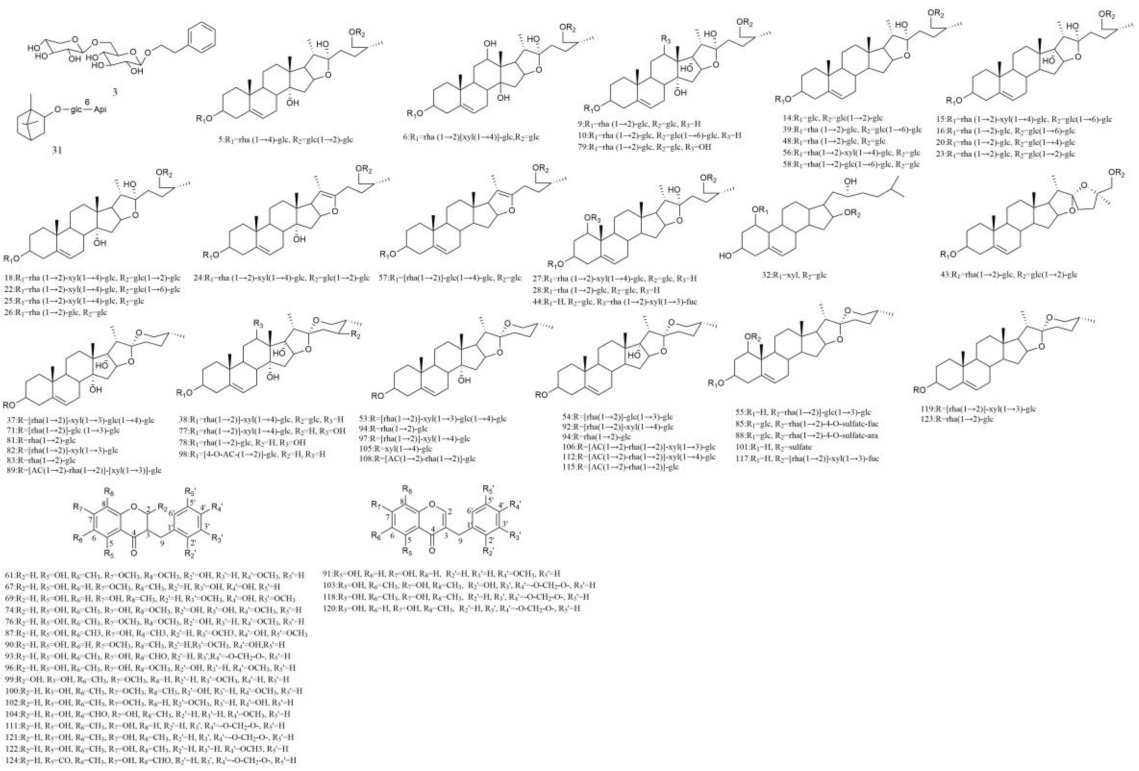

Figure S1. The chemical structure of EDG

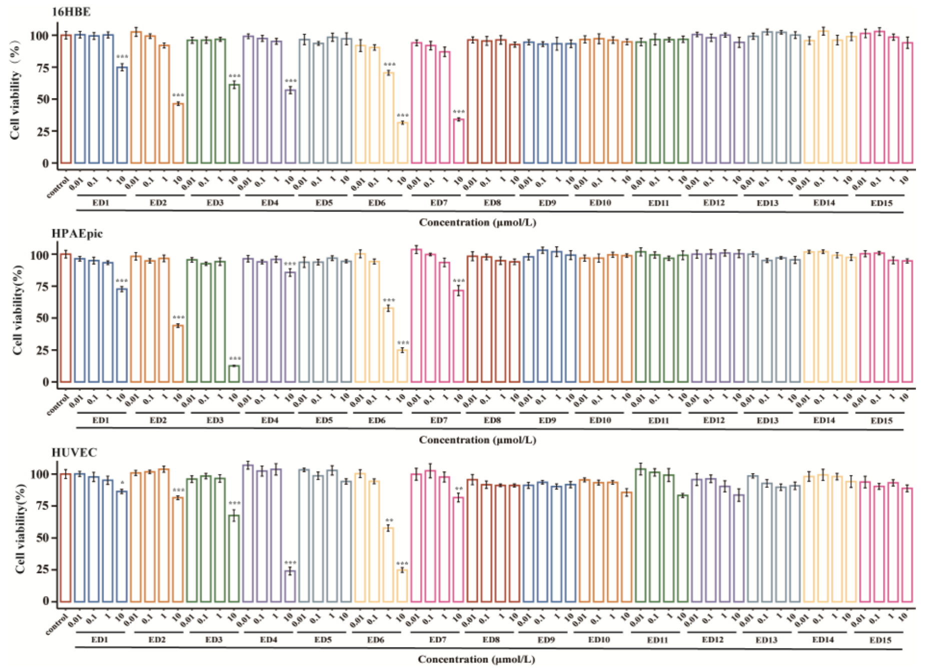

Figure S2. Effects of ED1-15 on 16HBE, HPAEpic and HUVEC cells viability. (16HBE, HPAEpiC and HUVEC cells were pretreated with ED1-15 at the concentration of 0.01-1 μM for 12 h. Cell viability was measured by the MTT assay. Results were obtained from three independent experiments and were presented as mean ± SEM. **P* < 0.05, ***P* < 0.01, ****P* < 0.001 vs control.)
